# Exploring the Roles of Aquaporins in Plant–Microbe Interactions

**DOI:** 10.3390/cells7120267

**Published:** 2018-12-11

**Authors:** Ruirui Wang, Min Wang, Kehao Chen, Shiyu Wang, Luis Alejandro Jose Mur, Shiwei Guo

**Affiliations:** 1 Jiangsu Provincial Key Lab of Solid Organic Waste Utilization, Jiangsu Collaborative Innovation Center of Solid Organic Wastes, Educational Ministry Engineering Center of Resource-Saving Fertilizers, Nanjing Agricultural University, Nanjing 210095, China; 2017203055@njau.edu.cn (R.W.); minwang@njau.edu.cn (M.W.); 2018103104@njau.edu.cn (K.C.); 14116303@njau.edu.cn (S.W.); 2 Institute of Biological, Environmental and Rural Sciences, Aberystwyth University, Aberystwyth SY23 3DA, UK; lum@aber.ac.uk

**Keywords:** aquaporins, plant–microbe interaction, water homeostasis, solute transport, signaling

## Abstract

Aquaporins (AQPs) are membrane channel proteins regulating the flux of water and other various small solutes across membranes. Significant progress has been made in understanding the roles of AQPs in plants’ physiological processes, and now their activities in various plant–microbe interactions are receiving more attention. This review summarizes the various roles of different AQPs during interactions with microbes which have positive and negative consequences on the host plants. In positive plant–microbe interactions involving rhizobia, arbuscular mycorrhizae (AM), and plant growth-promoting rhizobacteria (PGPR), AQPs play important roles in nitrogen fixation, nutrient transport, improving water status, and increasing abiotic stress tolerance. For negative interactions resulting in pathogenesis, AQPs help plants resist infections by preventing pathogen ingress by influencing stomata opening and influencing defensive signaling pathways, especially through regulating systemic acquired resistance. Interactions with bacterial or viral pathogens can be directly perturbed through direct interaction of AQPs with harpins or replicase. However, whilst these observations indicate the importance of AQPs, further work is needed to develop a fuller mechanistic understanding of their functions.

## 1. Introduction

Plants are constantly exposed to a multitude of microorganisms, with which they can form interactions with negative or positive consequences. Positive interactions are exemplified by symbiotic microorganisms which are beneficial for plant growth or activate natural defences. Negative interactions involve pathogens and can lead to disease. Many studies have focused on the mechanisms of plant–microbe interactions in terms their physical, biochemical, and molecular aspects, such as water availability [1,2], nutrients [3,4,5,6], root exudates [7,8,9], or signaling [10,11,12,13] aspects. These have established that water and nutrients play fundamental roles in the establishment of plant–microbe interactions.

The maintenance of water homeostasis is critically important for plants to sustain cellular and functional homeostasis during various growth conditions. Water can flow along cell wall structures (apoplastic path) or from cell to cell, along plasmodesmata (symplastic path) and both across the cell membrane in the root or in the leaves. Plant aquaporins, as channel proteins, are located in the plasma membrane and cytosolic regions, and play key regulatory roles in plant water transport [14,15,16,17,18,19]. In addition to transporting water, aquaporins (AQPs) can control the transcellular movement of small neutral molecules, such as CO_2_ [20,21,22], ammonia [23], hydrogen peroxide [24,25,26], and metalloids (boric acid, silicon, antimonite, arsenite) [27,28,29], or function as gated ion channels under certain conditions [30,31,32]. Given these roles, AQP influence growth regulation, hydraulic regulation, nutrient acquisition and translocation, and carbon fixation in roots and leaves [15,33,34,35,36,37], and are also involved in plant responses to stresses [26,38,39,40,41].

The specific roles or mechanisms of plant AQPs in plant–microbe interactions have been largely unexplored. Most studies have characterized the activities and gene expression of AQPs in plants in both positive and negative plant–microbe interactions. For instance, AQPs’ genes are up-regulated by mycorrhizal colonization in the roots of *Medicago truncatula* [42,43]. *Candidatus* Liberibacter asiaticus infection repressed the expression of genes encoding AQPs, while another was induced in citrus stems [44] or increased in pepper leaves after *Phytophthora capsici* infection [45]. Considering the diversity of AQPs, plants, and microbes, it is necessary to consider their diversity, cellular localization, and roles in different plant–microbe interactions. These are our aims in this review.

## 2. Plants’ Aquaporin Diversity and Function

AQPs are channel proteins belonging to the Major Intrinsic Protein (MIP) superfamily, and play an important role in plant–water relations. More than 100 AQPs have been discovered in plants, which now comprise a large and diverse protein family [35]. These are mainly clustered into five phylogenetic subfamilies depending on the plant species, membrane localization, and amino acid sequence. These classes are plasma membrane intrinsic proteins (PIPs), tonoplast intrinsic proteins (TIPs), nodulin 26-like intrinsic proteins (NIPs), small basic intrinsic proteins (SIPs), and uncategorized X intrinsic proteins (XIPs) [46,47,48].

PIPs have mainly been identified in the plasma membranes, and exist in two further subgroups: PIP1s and PIP2s [49]. PIPs function as the transporters of water, glycerol, H_2_O_2_, and carbon dioxide. In general, all PIP2 proteins exhibit high water-channel activity, whereas PIP1 proteins are often inactive or have low activity [50,51,52]. PIP2 efficiently transports water, mainly within the cell-to-cell pathway [53]. Although PIP1 showed poor aquaporin activity, PIP1–PIP2 interaction enhances water permeability. Co-expression of ZmPIP1;2 and ZmPIP2;5 resulted in increased water-channel activity [54]. PIPs are expressed ubiquitously throughout the plant and PIP1 and PIP2 can be co- or differentially regulated depending on the conditions, such as water supply and osmotic conditions [55,56], light [57], temperature [58,59], or hormones [59,60,61,62].

TIPs are the most abundant AQPs in the tonoplast [35]. AtTIP1;1 of *Arabidopsis thaliana* was the first identified plant water channel [63]. Due to the abundance of these AQPs in the tonoplast, the tonoplast has much higher water permeability [64]. TIPs, apart from its transport function (glycerol, urea, and ammonia) [65,66], is indispensable for growth under environmental stress [58,66].

NIPs are found in plasma membranes or the endoplasmic reticulum. Unlike PIPs or TIPs, NIP expression is restricted to defined cell types or tissues [67]. For example, NIPs are expressed during nodules formation, and play an important role in transporting water between the bacteria and the host plant. NIPs can be divided into four paralogous clades, NIP-1 to NIP-4, with different functions [68]. Nodulin 26 (Nod26), belonging to NIP-1 proteins, is considered to be a major integral protein of the symbiosome membrane, which surrounds the nitrogen-fixing bacteria with legume:rhizobia symbiosis. Compared to PIPs and TIPs, NIPs are less able to transport water, but confer higher permeability onto small organic molecules and mineral nutrients. In particular, they mediate the transport of boron (B), silicon (Si), selenium (Se), or arsenic (As) [69,70,71,72]. In addition, NIPs can also transport glycerol, ammonia, H_2_O_2_, and other solutes between the plant and bacterial symbionts [73,74,75,76,77]. NIP can too be considered as a novel marker of mycorrhizal status during arbuscular mycorrhizae (AM) symbiosis [78].

SIPs were localized in the endoplasmic reticulum (ER) and have been shown to facilitate water transport with different solute permeability compared to other AQPs [79]. SIPs are not structurally and functionally well-characterized. Plants’ SIPs may possibly be involved in tolerance to various stresses, such as hydrogen peroxide [80], B-regulation [81], and osmotic stress [82]. Interestingly, like XIPs, SIPs also have an evolutionary link between the plants and fungi [83]. Much work is needed to clarify the specific physiological role of SIPs.

XIPs generally sit at the plasma membrane and are expressed on the entire cell surface [84]. XIPs have been characterized in protozoa, fungi, and some non-vascular and vascular plants. However, the functional roles of XIP in plants remain poorly identified. Studies have suggested that the XIP subfamily works as a multifunctional channel not highly permeable to water but which favors larger, uncharged solutes [48,84,85]. XIPs can also be transcriptionally regulated during AM symbiosis, and the potential role of XIPs remain obscure [78].

## 3. Aquaporins in Plant–Microbe Interactions

Plants are hosts to a multitude of microorganisms. In positive interactions, microorganisms can enhance the productivity and performance of its host plants and include rhizobia, mycorrhizae, and plant growth-promoting rhizobacteria (PGPR). They mostly contribute to promote the uptake of nutrients and water or improve plant tolerance [86,87,88,89,90,91,92], notwithstanding little negative impacts of AM interactions on crop performance [93]. Alternatively, numerous pathogenic fungi, bacteria, and viruses can decrease plant productivity in direct or indirect ways [94,95,96]. AQPs have currently established positive roles on plants in both positive and negative plant–microbe interactions. The aquaporins’ isoforms considered in this article are summarized in Table 1.

### 3.1. Positive Plant–Microbe Interactions

#### 3.1.1. Rhizobia

A well-characterized positive plant–microbe interaction is rhizobium-legume symbiosis. Legumes can establish symbioses with the nitrogen gas (N_2_)-fixing bacteria rhizobia, which leads to the formation of the root nodule [113]. N_2_ in the atmosphere is unavailable for direct use by most plants. Symbiosis can convert N_2_ to inorganic ammonium. N_2_-fixing symbiosis is significantly important for the nitrogen’s environmental-friendly input in both agricultural and natural ecosystems. In rhizobium-legume symbiosis, AQPs, especially nodulin 26-like intrinsic proteins, play an important regulatory role in nitrogen absorption and assimilation.

NIP channels likely have a potential multifunctional role in the metabolism and osmoregulation in nodules of rhizobium-legume symbiosis. The most important role of nodulin 26 in rhizobium-legume symbiosis is nitrogen fixation, including NH_3_ transport and NH_4_^+^ assimilation.

**Figure 1:**
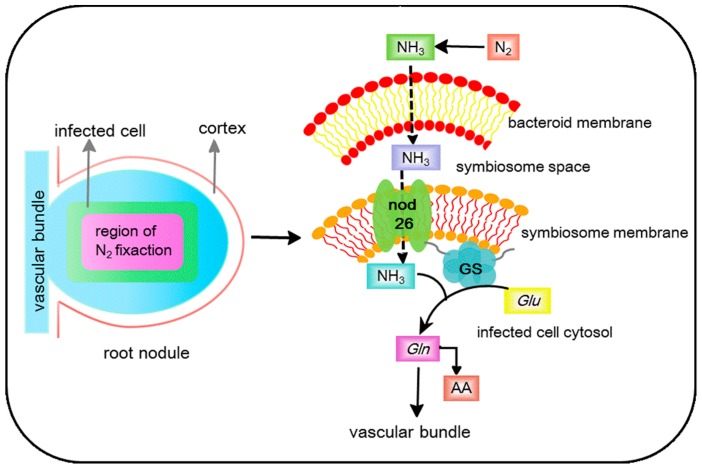
Root nodules showing regions of active nitrogen fixation, and a model of nitrogen efflux and assimilation with the interaction of nodulin 26/glutamine synthetase (GS). Fixed nitrogen within the symbiosome space can be transported by the nodulin 26. GS which bind to the C-terminal domain of nodulin 26 serves as a site in the symbiosome membrane for rapid assimilation ammonia, and then gets released into the infected cell’s cytosol. GS, glutamine synthetase; Glu, glutamate; Gln, glutamine; AA, amino acid.

The assimilation of symbiotes fixes nitrogen to the plant cytosol, as the symbiosome membrane acts as a barrier. Nodulin 26 is an ammoniaporin [76], and there is a high concentration of this on the symbiosome membrane, which facilitates NH_3_/NH_4_^+^ transport in a bidirectional manner [114]. Indeed, Nodulin-26 possesses a fivefold higher preference for ammonia compared to water [76]. The direction of transport would depend on the concentration gradient of NH_3_ across the symbiosome membrane. Upon transport to the infected cell cytosol, ATP-dependent glutamine synthetase (GS) can promote the ammonium ion to be assimilated into organic form [115]. This process could be accelerated by the combination of soybean nodule cytosolic GS with the nodulin 26 carboxyl terminal domain [116]. The association of GS with nodulin 26 can facilitate rapid nitrogen assimilation, preventing the accumulation of free ammonia in the cytosol nodulin 26, which has a potential interplay with GS to mediate ammonia efflux and assimilation (Figure 1).

Additional roles for AQP in rhizobium-legume symbiosis have been suggested for TIP1. During root nodule differentiation in barrel clover (*Medicago truncatula*), a TIP1 homolog was shown to be transiently retargeted from the tonoplast to the symbiosome membrane [97]. Further, the re-targeting of TIP1g is important for the distribution and maturation of symbiosomes in infected cells, which can probably be achieved by increasing the availability of water [97].

#### 3.1.2. Mycorrhizal

Mycorrhizae represent a very common symbiotic interaction between soil fungi and plant roots. The plant interface with mycorrhizal fungi may be intracellular (as with arbuscular mycorrhizae (AM)) or extracellular (as with ecto-mycorrhizae (EM)). In each case, mycorrhizal fungal mycelia improve nutrient status and water relations for their hosts, whereas the plant root provides carbon metabolites for nutrient assimilation. AQPs play an important role in both water and nutrient exchange in plant-mycorrhizal symbiosis [117]. Water conservation and absorption are two main mechanisms in plant-mycorrhizal symbiosis to cope better with stressful environmental conditions, such as drought, flooding, cold, or salinity [98,101,103,104,118]. To improve water relations, mycorrhizal symbioses can actively modify the function and gene expression of plant aquaporins to influence conditions of relative apoplastic water flow [42,119,120,121,122]. The regulation of plant AQPs seems to differ between the plant and fungal species that are involved in the symbiosis.

##### Mycorrhizal: AM Symbiosis

Arbuscular mycorrhizae are capable of establishing symbiotic relationships with many plants [123]. During the formation of the AM symbiosis, the plant plasma membranes undergo extensive morphological alterations that closely surround the fungal hyphae, resulting in an increase in the outer plant cell surface area [124]. As a result, symbiont can acquire nutrients (mainly phosphates) and water more efficiently than the root alone [125]. AQPs promote the morphological alterations of plants to increase symbiotic efficiency. The tobacco plasma membrane aquaporin NtAQP1 allows for CO_2_ passage and contributes to plant growth promotion [126]. Further, AM symbiosis indeed results in altered rates of water movement into, through, and out of the host plants [127], and also modifies root hydraulic conductivity under specific stressed conditions [98,103,128,129]. These events have been linked to AQPs. Compared to non-AM plants, AM plants significantly enhanced relative apoplastic water flow [128] and induced change in the expression of aquaporin-encoding genes, such as TIPs [119,120], PIPs [42,130], and NIPs [42]. The amount of PIP2 protein and its phosphorylation status strongly contributed to the regulation of root hydraulic properties by AM symbiosis in *Phaseolus vulgaris,* and enhanced root hydraulic conductivity under drought, cold, and salinity stresses in AM plants [98].

##### Abiotic Stresses Tolerance

It is now well-established that AQPs are involved in the regulation of host plant tolerance to stress in AM symbiosis as a process that depends on both plant and fungal interactors. Porcel et al. [99] observed that mycorrhizal wild-type plants grew faster than antisense tobacco plants which targeted mycorrhizal NtAQP1 under drought stress. This was linked to reduced symbiotic efficiency of AM fungi, mostly due to the reduced expression of *PIP* genes [99]. Furthermore, water conservation is an important stress tolerance mechanism, and the downregulation of AQPs protects AM plants from drought stress. *PIP* genes from *Glycine max* (*GmPIP1, GmPIP2*) and *Lactuca sativa* (*LsPIP1, LsPIP2*) were down-regulated following AM (*Glomus mosseae*) infection under drought stress and AM plants accelerated the downregulation of these genes compared to non-AM plants [101]. This aligned with the conclusions of previous experiments, where they showed that the overexpression of PIPs in transgenic tobacco or *Arabidopsis* had a negative effect on plants, causing fast wilting under drought stress [131,132]. Equally, under non-stressed conditions, *PIP* gene overexpression improved plant vigor. The decreased expression of *PIP* genes during drought stress in roots of AM plants can be a regulatory mechanism to decrease membrane water permeability and limit water loss from cells [101,133].

If this is the case, the AM fungal species and PIPs diversity could influence the efficacy of the above resistance mechanism. This was shown when lettuce plants were colonized with the AM fungus, *Glomus intraradices* where they did not exhibit such down-regulation of *PIP* gene expression, and the roots had higher water permeability [101]. In a comparative study involving colonization by *G. intraradice* or *G. mosseae*, the former absorbed water much more efficiently [102]. Considering the role of PIP isoforms, Aroca et al. [98] analyzed four *PIP* genes in roots from *Phaseolus vulgaris* which either were or were not colonized by AM fungi, and subjected to drought, cold, or salinity. *PvPIP1;1*, *PvPIP1*;2, and *PvPIP1;3* expression showed differences in AM and non-AM plants according to the stress imposed. Different genes showed differential function and regulation by AM symbiosis under the different conditions. Notwithstanding such subtleties, the overall observation that AQPs seem to influence water permeability and nutrient flux more efficiently in AM plants with stress appears to be the overall rule.

##### Solute transport

The role of AQPs-mediated solute transport in AM is also important. Several of AM-regulated maize AQPs (ZmPIP1;3, ZmPIP2;2, ZmTIP1;1, ZmTIP1;2, ZmNIP1;1, ZmNIP2;1, and ZmNIP2;2) transport glycerol, ammonia, or H_2_O_2_, as well as water, which are vital to plant physiological performance under well-watered conditions [103]. Glycerol is also transported by tobacco NtTIPa [100], ZmNIP1;1, ZmTIP4;1 and ZmTIP4 of maize [103], OsTIP1;2, OsTIP3;2, and OsTIP4;1 from rice [96]. While the physiological function of glycerol in plants is unclear, glycerol is an important carbon source for pathogenic fungi [134] and may be important in establishing symbiotic relationships [135,136]. Thus, AQPs transporting glycerol from the plant to the microbe will facilitate AM symbiosis under sustained drought stress.

The regulation of nitrogen movement is also tightly related to AQPs in higher plants. The PIP, NIP, and TIP subfamilies can transport ammonia and urea to maintain their relative balance between the cytoplasm and vacuole [37,137,138]. Almost all TIP subclasses in *Arabidopsis* transport urea [139], as do ZmNIP2;1, ZmNIP2;4, and ZmTIP4;4 in maize [137]. This is of direct relevance to AM symbiosis, where inorganic nitrogen (ammonium and nitrate) is assimilated and transferred to the host plant [140], and urea plays a role as an intermediate solute [141,142]. Such suggestions by ZmTIP1;1 and ZmTIP1;2, which were regulated by the AM symbiosis, have been shown to transport both ammonia and urea [103].

Beyond nutrition, AQP could influence redox-mediated events. Superoxide-generating nicotinamide-adenine dinucleotide phophate (NADPH) oxidases located on the plasma membrane are major sources of apoplastic hydrogen peroxide (H_2_O_2_) production during AMF symbiosis. H_2_O_2_ accumulates in symbiont cells and around hyphal tips attempting to penetrate a host cell [143,144]. Such can reduce penetration by augmenting the plant’s innate defence response. However, the H_2_O_2_ could be removed from the interaction site by diffusion across the plasma membrane through AQP channels where it can be reduced by potent antioxidant systems [24]. For example, ZmTIP1;1 has been shown to transport H_2_O_2_ for detoxification under stress conditions [103]. The generation of reactive oxygens species is a feature which is common to many stresses and is a central event in plants’ responses to them, so the role of AQPs in the transport and relocation of H_2_O_2_ as a detoxification or a signaling mechanism needs to be studied further.

##### Mycorrhizal: EM Symbiosis

Another type of mycorrhizal interaction is ectomycorrhizal symbiosis. In this form of symbiosis, the fungal hyphae are extracellular to plant cells and closely surround the roots, or form a thick network known as a Hartig net between the epidermal and cortical cells [145,146]. EM fungi provide the host plant with mineral nutrients and water in exchange for carbon compounds from the host [147]. AQPs play a major role in these aspects of EM symbiosis.

As with AM fungi, AQPs regulate plant defences’ responses to facilitate EM symbiosis. Rather than crops (bean, soybean, or lettuce), the host plants of EM fungi are mostly perennial woody plants with strong seasonality, and EM symbiosis will enhance root water transport, which has been linked to increased AQP expression. This increased AQP has been shown to influence the rate of transmembrane water transport and, consequently, root hydraulic conductance, even in cell-to-cell pathways [35,106]. In poplar, *PttPIP2;5* was upregulated in EM roots compared to non-mycorrhizal seedlings, and this was more important in water stress conditions [104]. Similarly, expression of *PttPIP2.2* and *PttPIP2.4* was higher in mycorrhized poplar plants under drought stress [105]. Tarkka et al. [148] also found an increase in the expression of one *SIP* and five *PIP* genes in *Quercus robur* roots inoculated with the EM fungus. Some studies showed that the upregulation of *PIP* genes was correlated with higher root hydraulic conductance and increased water transport from the fungus to the intercellular EM root cells in poplar [104,105]. Such higher expression of *PIP* genes with higher root-specific conductance could partially compensate for certain plants having smaller root sizes which could otherwise limit water transport in dwarf plants [106]. However, the role for AQPs in roots’ EM symbiosis is not a universal feature of all host plants. In contrast to the poplar, inoculation of *A. muscaria* on Norway spruce roots had no effect on the expression of root AQPs. Also, two *PIP* genes of the *Picea glauca*-inoculated *Laccaria bicolor* were downregulated with enhanced root hydraulic conductance [107].

**Figure 2:**
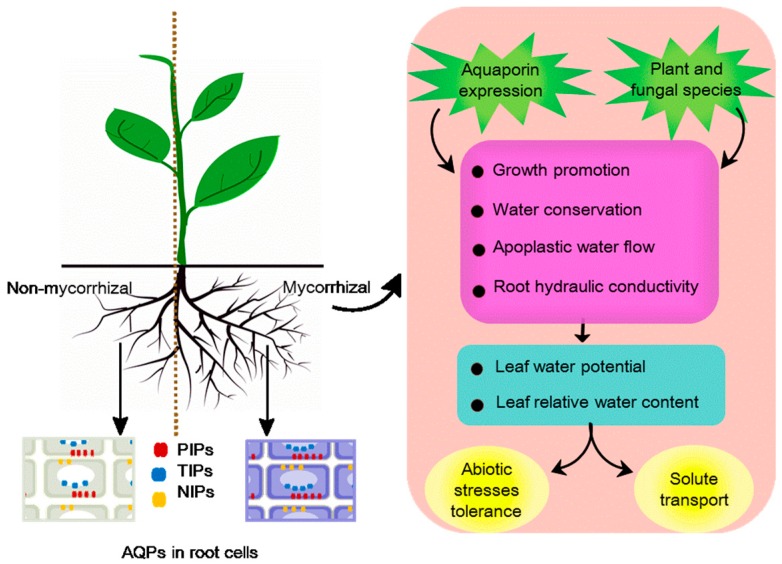
Model of the role of aquaporins (AQPs) in arbuscular mycorrhizae (AM)/ecto-mycorrhizae (EM) symbioses combined with plant–water relations and physiological activity. Mycorrhizal symbioses promote growth of mycorrhizal plants, with a larger root system (the right side of the root system). The amount of AOPs in mycorrhizal plant root cells (right side, blue) is generally more than that in non-mycorrhizal plant root cells (left side, gray). Plasma membrane intrinsic proteins (PIPs) and nodulin 26-like intrinsic proteins (NIPs) are in plasma membranes, and tonoplast intrinsic proteins (TIPs) are in the tonoplast (white oval). The regulation of plant AQPs contributes to growth promotion, water conservation, apoplastic water flow, and root hydraulic conductivity to maintain the relative water content and leaf water potential. Favorable water relation promotes solute transport and increases abiotic stress tolerance. Plant and fungal species could also influence the efficacy of the above resistance mechanism.

Leaving aside what could be exceptions to a general rule, the literature strongly supports the importance of AQPs in AM/EM symbioses in regulating plant–water relations (relative water content and leaf water potential) and physiology (morphology, nutrition, signaling molecules and other solutes), and these are summarized in Figure 2.

#### 3.1.3. Plant Growth-Promoting Rhizobacteria (PGPR) 

Plant growth-promoting rhizobacteria (PGPR) are naturally occurring soil bacteria inhabiting rhizospheres, which facilitate plant growth by improving plant productivity and immunity under normal and stress conditions [149]. PGPR has largely been documented to contribute to plant tolerance against drought and salinity [108,150]. Under such conditions, plants must decrease their cell water potential to continue to take up water to reduce the impact of increased salinity or water limitation. This clearly indicates a role for AQPs, but the literature suggests that the responses of host-microbiome interactions are specific and require further characterization [151].

Inoculation with *Azospirillum brasilense*, the nitrogen-fixing bacterial species, has been shown to result in the upregulation of *HvPIP2-1* in barley seedlings [108]. Maize plants inoculated with *Bacillus megaterium* has shown increased root hydraulic conductivity compared to uninoculated plants when exposed to salinity, and this was correlated with increased expression of *ZmPIP1;1* and *ZmPIP1;5* [109]. In a similar way, *Pantoea agglomerans* and *B. megaterium*-infected *Zea mays* roots showed upregulated *PIP2* and *ZmPIP1-1* expression regulated under salt stress conditions, which contributed to increased root hydraulic conductance in inoculated plants [152]. However, *ZmPIP2;1* was down-regulated in inoculated roots, although this was up-regulated by salt addition alone. This inoculation effect was not seen post-transcriptionally where ZmPIP1;2 accumulation occurred [109], and was likely to increase the water-use efficiency of PGPR-interacting plants [153]. In lettuce plants inoculated with *Pseudomonas mendocina*, the maintenance of relative water content during drought stress correlated with a downregulation of *PIP2*. Indeed, *PIP2* expression was significantly enhanced by *P. mendocina* only in plants grown under well-watered conditions [110].

### 3.2. Negative Plant–Microbe Interactions

**Figure 3:**
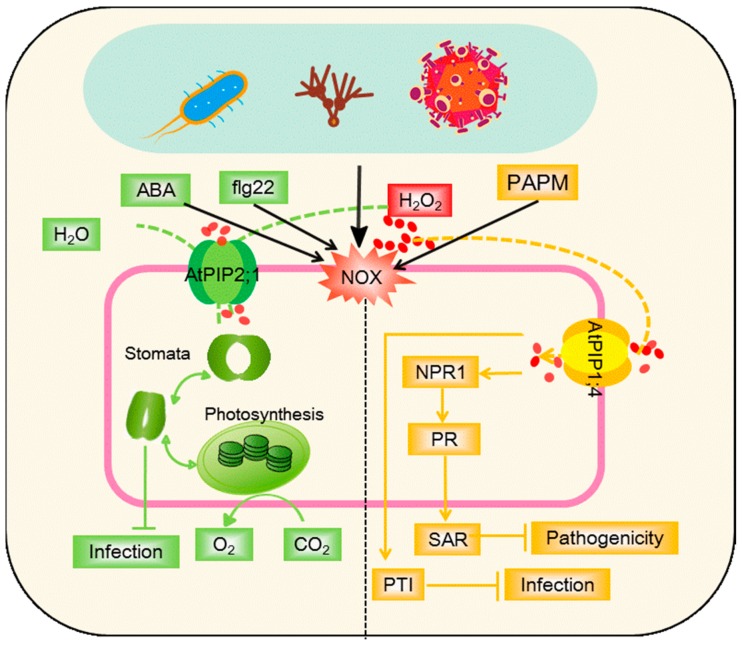
Model of AQPs-mediated linkage of apoplastic H_2_O_2_ to stomatal closure (green) and immunity pathways (systemic acquired resistance (SAR) and PAMP-triggered immunity (PTI)) (yellow). Apoplastic H_2_O_2_ can be induced by the pathogen or pathogen-associated molecular pattern (PAMPs, flg22), generated through the NADPH oxidase (NOX) activity and rapidly translocated into the cytoplasm under regulation by AtPIP2;1 or AtPIP1;4. Abscisic acid (ABA) and flg22 (a PAMP) activate AtPIP2;1 to facilitate transport of both water and H_2_O_2_, and promote stomatal closure to restrict bacterial invasion. Translocated H_2_O_2_ cooperates with SAR or PTI to repress the pathogenicity. ABA, abscisic acid; H_2_O_2_, hydrogen peroxide; PAMP, pathogen-associated molecular patterns; flg22, a 22-amino acid eubacterial flagellin-derived peptide; NOX, NADPH oxidase; PR, pathogenesis-related genes; NPR1, non-inducer of PR genes1; SAR, systemic acquired resistance; PTI, PAMP-triggered immunity.

The literature strongly supports the roles of AQP in various forms of symbiotic interactions, yet the roles of AQPs in negative plant–microbe interactions are largely unknown. Plant pathogens effectively utilize plant nutrients solely for their growth and survival to the detriment of their plant hosts. AQPs expression patterns, manipulated by the pathogen, could play a role in this, as could the dehydration in the host that is a common disease symptom. The roles of AQPs in such negative interactions are depicted in Figure 3.

In considering the effects of AQP on physiological activity linked to disease, stomata are crucial to the control of plants’ water status in response to pathogens’ attack, and are potential entry points for foliar pathogens (Figure 3, green). Plants have the capacity to close their stomata after the perception of pathogen-associated molecular patterns (PAMP) to restrict bacterial invasion [154]. Rodrigues et al. [25] proposed a model whereby flg22 (a 22-amino acid eubacterial flagellin-derived peptide, a PAMP) and abscisic acid (ABA) activated PIP2;1 through phosphorylation at a conserved site, to facilitate transport of both water and H_2_O_2_ to promote stomatal closure. H_2_O_2_ production in the stomatal guard cells appears to be elicited by both ABA and PAMP, which can be transported into the cytosol by AQPs to mediate stomatal movement, acting through PIP2;1 [25,155].

Transport of apoplastic H_2_O_2_ to the cytoplasm of plant cells has been demonstrated via AtPIP1;4 following challenges to the bacterial pathogen, *Pseudomonas syringae* [26] (Figure 3, yellow). Once within the cell, this H_2_O_2_, and therefore AtPIP1;4, plays a key role in plant immunity pathways. Thus, in the cytoplasm, the translocated H_2_O_2_ activates the PAMP-triggered immunity (PTI) pathway to resist pathogens. It also activates the biosynthesis of salicylic acid, which acts via the signaling component, NPR1 to establish systemic acquired resistance (SAR) [26]. PIP1;4 also plays a role on effector-triggered immunity (ETI), where effectors are delivered from the pathogen into the host as part of the pathogenic process. Certain plant genotypes can recognize the effectors to initiate ETI.

In bacterial pathogens, effector delivery is done via a type of secretion mechanism which includes the harpin protein hpa1. Hpa1 interacts with aquaporin PIP1;4 of *Arabidopsis* [156] and OsPIP1;3 of rice. Hpa1 interactions with *Arabidopsis* AtPIP1;4 mediates the transport of CO_2_ and H_2_O and contributes to plant growth enhancement. In particular, this CO_2_ transport is linked to increases in leaf photosynthesis rates. Therefore, plant sensing of a bacterial harpin protein is connected with photosynthetic physiology to regulate plant growth [156]. For viruses, TIPs can interfere with intracellular viral replication by interacting with a cucumber mosaic virus (CMV) replication protein in the host plant’s tonoplasts [157]. Interactome data provide important clues about the involvement of aquaporins in host–pathogen interactions.

As H_2_O_2_ transport is important in responses to challenges by a pathogen, the linked changes in AQP expression are also crucial in developing our understanding of the defence network. The citrus plants major intrinsic protein *CsMIPs* showed differential expressions when infected with *Candidatus* Liberibacter asiaticus (*Ca.* L. asiaticus), the causal agent of huanglongbing disease [158]. *Ca.* L. asiaticus infection repressed expression of PIP1A, PIP1-1, PIP1-3 genes, while it induced expression of the *SIP1A* gene to affect the water and nutrients status [44]. The expression of AQP could be very infection stage-specific and reflects different roles in a dynamic process. This was suggested by the expression pattern of *CaPIP1-1* in pepper leaves challenged with *Phytophthora capsici*, which were up-regulated in the stage of spore germination along with plant metabolism observed to change [112].

## 4. Conclusions and Perspectives

**Figure 4:**
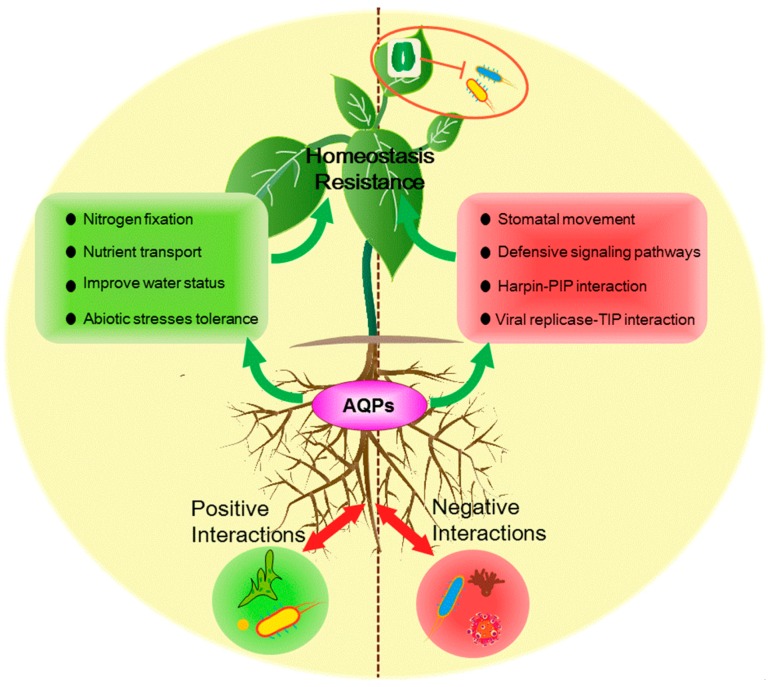
AQPs’ currently established positive roles in different pathways on plants in both positive and negative plant–microbe interactions. For positive plant–microbe interactions, AQPs mainly transport water and other physiological substances to maintain relative water content and leaf water potential by regulating specific AQP gene expression and activity under specific growth conditions. For negative plant–microbe interactions, AQPs use signaling in guard cells to adjust stomata and can interact with pathogen effectors or replicase, and can also induce resistance of plants by H_2_O_2_ signaling.

AQPs seem to play various roles in different plant–microbe interactions, as shown in Figure 4. AQPs mainly transport water and other physiological substances to maintain relative water content and leaf water potential by regulating specific AQP gene expression and activity under specific growth conditions. The above mechanism is most established for positive plant-microbe interactions. For negative plant-microbe interactions, AQPs use signaling in guard cells to adjust stomata and can interact with pathogen effectors or replicase, and can also induce resistance of plants by H_2_O_2_ signaling. In all, AQPs are responsible for maintaining water and nutrient homeostasis and increasing resistance against pathogen. Although numerous studies have elucidated the possible role of AQPs in plant–microbe interactions, detailed mechanisms of AQPs regulating plant–microbe interactions as a channel for other substances or signaling need further study. And attention should be focused on the following issues in the future:

1. AQP is a kind of channel, and future research should focus on identifying their capacity to transport mycelium, spores, or small secondary metabolites of pathogen.
2. Regulation of AQP by different plant–microbes interactions on complex processes and signaling pathways, as well as complex transcriptional, translational, and posttranscriptional factors.
3. The role of microbe APQs in regulation of the plant–microbe interaction.

## Figures and Tables

**Table 1:** Plant aquaporins (AQPs) involved in plant–microbe interactions.

Microbes		Host	AQP Isoform	References
Positive plant–microbe interactions	Rhizobia	Legume	TIP1g; NIPs	[76,97]
	Mycorrhizae: AM	Bean Tobacco Soybean Lettuce Maize Rice	PvPIP NtAQP1; NtTIPa GmPIP2 LsPIP ZmPIP; ZmTIP; ZmNIP OsTIP	[98] [99,100] [101] [101,102] [103] [61]
	Mycorrhizae: EM	Poplar Olive White spruce	PttPIP OePIP PgPIP	[104,105] [106] [107]
	PGPR	Barley Maize Soybean Lettuce	HvPIP ZmPIP GmPIP LsPIP	[108] [109] [101] [110]
Negative plant–microbe interactions	*Candidatus* Liberibacter *Phytophthora capsici*	Citrus Pepper	CsPIP; CsTIP; CsNIP CaPIP	[44,111] [112]
